# Comprehensive Identification and Expression Profiling of Epidermal Pattern Factor (*EPF*) Gene Family in Oilseed Rape (*Brassica napus* L.) under Salt Stress

**DOI:** 10.3390/genes15070912

**Published:** 2024-07-12

**Authors:** Shanshan Wang, Wei Wang, Jingdong Chen, Heping Wan, Huixia Zhao, Xiaoyun Liu, Xigang Dai, Changli Zeng, Danyun Xu

**Affiliations:** Hubei Engineering Research Center for Protection and Utilization of Special Biological Resources in the Hanjiang River Basin, College of Life Science, Jianghan University, Wuhan 430056, China; wang_shanshan0044@163.com (S.W.); jhunww@jhun.edu.cn (W.W.); cjd19951226@126.com (J.C.); wanheping@jhun.edu.cn (H.W.); zhaohuixia@jhun.edu.cn (H.Z.); liuxiaoyun@jhun.edu.cn (X.L.); xg_dai@163.com (X.D.); zengchangli@jhun.edu.cn (C.Z.)

**Keywords:** *Brassica napus*, *EPFs*, salt stress, stress tolerance from non-living factors

## Abstract

Rapeseed is a crucial oil crop globally, and in recent years, abiotic stress has increasingly affected its growth, development, yield, and quality. Salt stress is a significant abiotic factor that restricts crop production. The *EPF* gene family is vital in managing salt stress by controlling stomatal development and opening, which reduces water loss and increases plant salt tolerance. To explore the features of the *EPF* gene family in *Brassica napus* and their expression under salt stress, this study utilized *Arabidopsis* EPF protein sequences as seed sequences, including their PF17181 and PF16851 domains. A total of 27 members of the *EPF* gene family were detected within the rapeseed genome. The study examined the physicochemical properties, gene structure, phylogenetic relationships, and collinearity of *BnEPFs*. Through transcriptomes, we employed the qPCR method to determine the relative expression levels of *BnEPF* genes potentially associated with rapeseed stress resistance under both non-salt and salt stress conditions. Subsequently, we assessed their influence on rapeseed plants subjected to salt stress. During salt stress conditions, all *BnEPF* genes displayed a downregulation trend, indicating their potential impact on stomatal development and signal transduction pathways, consequently improving rapeseed’s resistance to salt stress. The study findings establish a basis for exploring the roles of *BnEPFs* and offer candidate genes for breeding stress-resistant varieties and enhancing the yield in rapeseed.

## 1. Introduction

Rapeseed (*Brassica napus* L.) stands as a vital global oil crop renowned for its seeds’ high nutritional content and abundant in fatty acids and vitamins, serving diverse industrial purposes [1,2,3,4]. Due to the low production resulting from limited planting areas, China mainly relies on imports for oilseed crops. By utilizing saline–alkali soils to cultivate salt-tolerant rapeseed, especially in the idle lands of the northern and coastal regions of China, the over-reliance on the international market can be alleviated. Rapeseed is susceptible to salt stress, which leads to reduced yields, hence ensuring that a consistent rapeseed cultivation area holds immense importance for the sustainable progress of global agriculture.

Stomata function as the primary conduits for both water loss and carbon dioxide absorption. The harmonization of carbon assimilation and transpiration hinges upon the delicate adjustment of stomatal aperture [5]. Upon exposure to abiotic stress, plants enhance their resilience by modulating stomatal aperture to minimize water loss and regulate carbon dioxide uptake [6]. In reaction to alterations in the external surroundings, plants can control CO_2_ intake and water loss by regulating stomatal aperture or density. This contributes significantly to bolstering plant resilience against stressors like drought and high temperatures. Changes in stomatal traits can, to some extent, enhance photosynthetic performance and water utilization efficiency in plants [7,8,9].

The EPIDERMAL PATTERNING FACTOR (*EPF*) family consists of a group of cysteine-rich secreted peptides that are pivotal in regulating stomatal development, thus facilitating plant growth, development, and stress responses [10]. Some genes in this family are involved in the early development of stomata and regulate stomatal density in plants [11,12]. Currently, the *EPF* gene family has received considerable attention and has been studied across various plant species such as *Arabidopsis thaliana* [11,13], *Physcomitrella patens* [14], barley (*Hordeum vulgare*) [15], rice (*Oryza sativa*) [16], and maize (*Zea mays*) [16]. Knocking out the *OsEPFL9* gene or using RNA interference in rice can significantly reduce the stomatal density in its leaves, while overexpressing *OsEPFL9* can significantly increase the stomatal density in rice leaves [17]. However, studies have also demonstrated that heterologous overexpression of barley *HvEPF1* [15], rice *OsEPF1* and *OsEPF2* [16], and wheat *TaEPF1B* and *TaEPF2D* [18] significantly reduces stomatal density in *Arabidopsis thaliana,* thereby improving water use efficiency. In studies on rice (*Oryza sativa*) [17], barley (*Hordeum vulgare*) [15], potato (*Solanum tuberosum*) [19], and poplar (*Populus*) [20], similar results have been obtained, showing that *EPF* genes can reduce stomatal density and improve water use efficiency. This indicates that *EPF* genes can regulate stomatal development processes, thereby modulating stomatal density and influencing plant drought resistance and water use efficiency. Additionally, research also indicates that *EPF* genes can participate in regulating the plant floral structure and leaf morphology and mediating processes such as pollen tube elongation during morphogenesis [21,22]. While the *EPF* gene family has been investigated in other plant species, its precise role in conferring salt tolerance in rapeseed remains incompletely elucidated. However, the formation and regulation of stomata significantly influence plant salt tolerance. The *EPF* family fulfills a pivotal role in stomatal development because stomatal aperture directly affects the absorption and exclusion of salt by plants, thereby influencing plant growth and survival under salt stress conditions. Therefore, the *EPF* family may influence plant salt tolerance by regulating stomatal development and function. Future research may further elucidate the specific mechanisms of the *EPF* family in plant salt tolerance.

This study performed a comprehensive genome-wide identification of the *EPF* gene family in *B. napus*, compared its collinearity with *EPF* family members in Chinese cabbage (*Brassica rapa*) and cabbage (*Brassica oleracea*), analyzed its gene and protein structures, and examined its evolutionary relationships. Additionally, it analyzed the spatiotemporal expression profiles of these genes and their reactions to non-living stressors using transcriptomic data and qRT-PCR. This research aims to establish the groundwork for further understanding the roles of the *EPF* gene family in the growth and development of *B. napus*, especially in response to salt stress.

## 2. Methods

### 2.1. Identification and Chromosome Localization of BnEPF Family Members

Sequence files for ZS11 (a variety of *B. napus*), cabbage (*B. rapa*), and kale (*B. oleracea*) were retrieved from the BRAD database (BRAD: http://brassicadb.cn/#/, accessed on 25 April 2024) [23]. Eleven AtEPF protein sequences from the *A. thaliana* genome database (TAIR: https://www.arabidopsis.org/, accessed on 25 April 2024) were used as seed sequences. These sequences were then employed in a BLASTp search to identify potential BnEPF proteins within the complete protein sequences of *B. napus*, using an e-value cutoff of less than 1 × 10^−10^ [24]. Conserved domain files PF17181 and PF16851 were downloaded from the Pfam database and used with HMMER 3.1 software (http://www.hmmer.org/, accessed on 25 April 2024) to identify potential BnEPF proteins. PF17181 targeted EPF1, EPF2, and EPFL1 through EPFL8, while PF16851 targeted EPFL9 [25,26]. These results were combined with BLAST search results to identify hypothetical BnEPF proteins. The predicted protein sequences were uploaded to the NCBI-CDD website (https://www.ncbi.nlm.nih.gov/cdd/, accessed on 25 April 2024) for further verification, leading to the final identification of 27 members of the *BnEPF* gene family. The *BnEPFs* were named based on their chromosomal positions, resulting in them being designated as *BnEPF1* through *BnEPF27*. The physical and chemical properties of BnEPF proteins were predicted using the ProtParam tool available on the ExPASy website [27], and the subcellular localization of BnEPF proteins was predicted using the CELLO v.2.5 tool [28]. The chromosomal locations of *BnEPFs* were obtained from downloaded annotation files and visualized using TBTools 1.047 [29].

### 2.2. Phylogenetic Assessment

The EPF protein sequences from *A. thaliana*, *B. napus*, *B. rapa*, and *B. oleracea* were uploaded into MEGA 11 software. Using the neighbor-joining (NJ) method with 1000 bootstrap repetitions, a phylogenetic tree was constructed [30]. The resulting phylogenetic tree was further refined using the online platform iTOL (https://itol.embl.de/, accessed on 25 April 2024) [31].

### 2.3. Prediction of Gene Structure, Conserved Motifs in prOTEins, and Cis-Regulatory Elements in Promoters

Gene structure and CDS/UTR data for members of the *BnEPF* gene family were retrieved from the NCBI-CDD and Pfam databases. Conserved motifs in the BnEPF proteins were identified using the MEME Suite version 5.5.1 [32]. Cis-acting elements within the 2000 bp upstream regions of the *BnEPF* promoters were predicted using the PlantCARE website [33]. The visualization of all information was performed using TBTools.

### 2.4. Collinearity Analysis of EPF Genes

The collinear relationships among *EPFs* within the genomes of *B. napus*, *B. rapa*, and *B. oleracea*, and intra-genomic collinearity within *B. napus*, were analyzed using MCScanX software [34]. The visualization of collinear relationships was conducted using TBTools. Ka/Ks values were determined using the KaKs_calculator 3.0 software [35].

### 2.5. Prediction of the Interaction between miRNA and BnEPFs

The CDS sequences of *BnEPFs* were submitted to the psRNATarget website (https://www.zhaolab.org/psRNATarget/analysis?function=2/, accessed on 26 April 2024) for analysis [36], in combination with miRNA sequences of *B. napus* available on the same site. This facilitated the investigation of targeting relationships between miRNAs and *BnEPFs*. Data visualization was executed by the alluvial plot tool on the Bioinformatics website (https://www.bioinformatics.com.cn/plot_basic_alluvial_plot_017, accessed on 26 April 2024).

### 2.6. Analysis of Transcriptome-Wide Expression Patterns

To investigate how *BnEPF* genes respond to different exogenous hormone treatments and abiotic stress conditions, we queried the gene IDs of *BnEPFs* in the BnIR database [37]. This allowed the retrieval of Transcript per Kilobase per Million mapped reads (TPM) data for *B. napus* ZS11 under a control (CK) condition, as well as under treatments with 10 μmol·L^−1^ exogenous hormones, including IAA (growth hormone), ACC (ethylene), GAs (gibberellin), ABA (abscisic acid), TZ (trans-zeatin), JA (jasmonic acid), and BL (brassinolactone), measured after 6 h. Additional data were gathered for a control (CK) condition and stress treatments including salt (200 mmol·L^−1^ NaCl), drought (exposure to airflow for 1 h), freezing (recovery to 25 °C after 3 h at −4 °C), cold (4 °C), heat (recovery to 25 °C after 3 h at 38 °C), and osmotic stress (300 mmol·L^−1^ mannitol) measured after 24 h. The data were processed by calculating log_10_ (TPM + 1) and were visualized using TBTools 1.047 software.

### 2.7. Materials and Treatment

The experimental material used was *B. napus* cv. Jia 917. Two clean germination boxes were prepared, each lined with a layer of clean gauze and filled with 1 L of Hoagland nutrient solution to ensure that the solution saturated the gauze. One germination box served as the non-salt treatment (NS: NaCl: 0%), while the other was used for the salt treatment (TS: NaCl: 0.8%). Jia 917 seeds were placed in the germination boxes and cultured for 7 days under a photoperiod of 16 h light and 8 h dark. After the 7-day period, three healthy and uniform seedlings were selected from each treatment. Photographs were taken, and the root length and hypocotyl length were measured using ImageJ V1.8.0.112 software [38]. The harvested seedlings were preserved in liquid nitrogen for subsequent qRT-PCR experiments. The experiment was conducted at the Breeding Factory of the School of Life Sciences, Jianghan University, Wuhan, Hubei Province, China (30°30′ N, 114°9′ E).

### 2.8. qRT-PCR Analysis of EPF Gene Expression Patterns

RNA extraction was conducted from rapeseed samples (Jia 917, Provided by the National Rapeseed Engineering Center of Huazhong Agricultural University) using the Tiangen RNAsimple Total RNA Kit (Tiangen Biotech, Beijing, China). Samples were subjected to reverse transcription using the HiScript^®^ II Q Select RT SuperMix with gDNA remover to obtain cDNA. qRT-PCR primers were generated with the assistance of Primer 6.0 software (Table 1), and qRT-PCR analysis was conducted using the AriaMx Real-Time PCR System (Agilent Technologies, Santa Clara City, CA, USA). The qRT-PCR reaction steps were as follows: initial denaturation at 95 °C for 30 s, heating at 95 °C for 5 s, cooling at 55 °C for 1 min, over a span of 40 cycles. Each sample underwent testing with three biological replicates, and the relative gene expression was determined using the 2^−∆∆CT^ method [39].

### 2.9. Statistical Procedures Were Applied for Data Analysis

qRT-PCR data were processed and plotted using SPSS 22.0, Origin Lab 2023, and GraphPad Prism 9.0.

## 3. Results

### 3.1. Identification and Characterization of EPFs in Rapeseed

In order to ascertain *EPFs* in oilseed rapeseed, we used eleven AtEPF protein sequences to perform BlastP and HMM searches within the genome of rapeseed “ZS11”. Then, sequences with incomplete structural domains were subsequently removed using the NCBI-CDD tool. After this refinement, a total of 27 BnEPF proteins were confirmed, sequentially named BnEPF*1* through BnEPF27, in accordance with the chromosomal location of their encoding genes.

**Figure 1:**
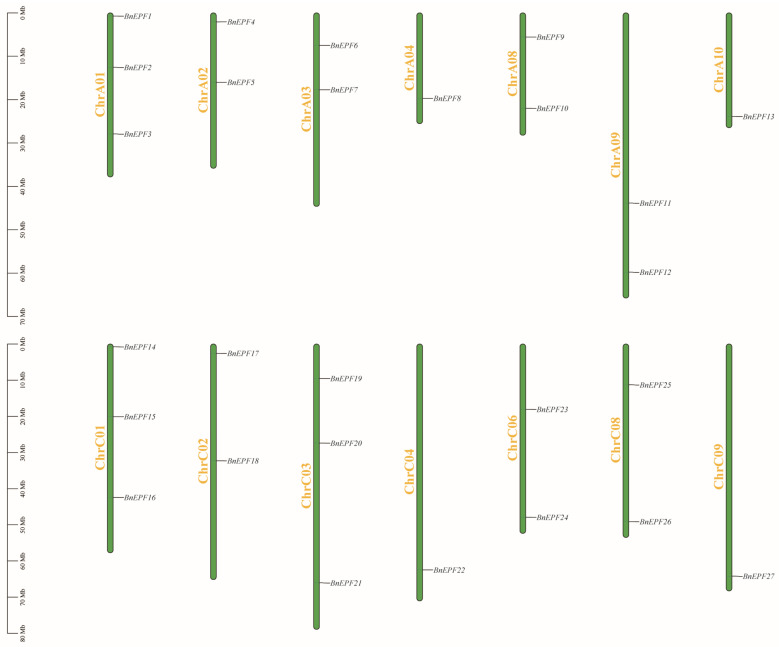
The arrangement of *BnEPFs* across the chromosomes of *B. napus*. Each chromosome’s name is indicated on the left side of the respective green bar, with gene names presented on their right. The scale on the far left denotes the physical position in megabases (Mb).

These 27 genes were distributed across 14 chromosomes (Figure 1 and Supplementary File S1). Specifically, chromosomes ChrA01, ChrC01, and ChrC03 each hosted three *BnEPFs*, while chromosomes ChrA02, ChrA03, ChrA08, ChrA09, ChrC02, ChrC06, and ChrC08 each had two *BnEPFs*. Chromosomes ChrA04, ChrA10, ChrC04, and ChrC09 each contained one BnEPF. Conversely, no *BnEPFs* were possessed by chromosomes ChrA05, ChrA06, ChrA07, ChrC05, and ChrC07.

### 3.2. Physical and Chemical Characteristics along with Subcellular Localization Prediction of BnEPF Proteins

The length of the aforementioned BnEPF proteins ranged from 56 to 150 amino acids (Supplementary Files S1 and S2). Their relative molecular weight (MW) ranged between 6.25 kDa and 16.62 kDa. The theoretical isoelectric points (PI) of these proteins ranged from 6.88 to 10.25. The grand average of hydropathy (GRAVY) for all BnEPFs was predicted to fall within the range from −0.72 to 0.50, suggesting significant hydrophilicity among BnEPF proteins. According to subcellular localization predictions, the majority of BnEPF proteins (From BnEPF1 to BnEPF6, from BnEPF8 to BnEPF19, and from BnEPF21 to BnEPF27) were located in the extracellular space, while a small fraction of BnEPF proteins (BnEPF7, BnEPF20) were found in the nucleus.

The secondary structure prediction for BnEPF proteins indicated the presence of three distinct structural elements as follows: α-helix (Hh), extended backbone (Ee), and random coil (Cc) (Supplementary File S3). Among them, the Cc conformation predominated, with a distribution range between 57.43% and 79.41%. The proportion of the Hh conformation was moderate, ranging from 1.79% to 27.97%, while the Ee conformation has the lowest proportion, ranging from 6.06% to 20.48%. Additionally, tertiary structure prediction showed that the BnEPF protein was predominantly composed of Cc conformations, which was consistent with the secondary structure prediction derived from homology modeling.

### 3.3. Evolutionary Analysis of the EPF Family

**Figure 2:**
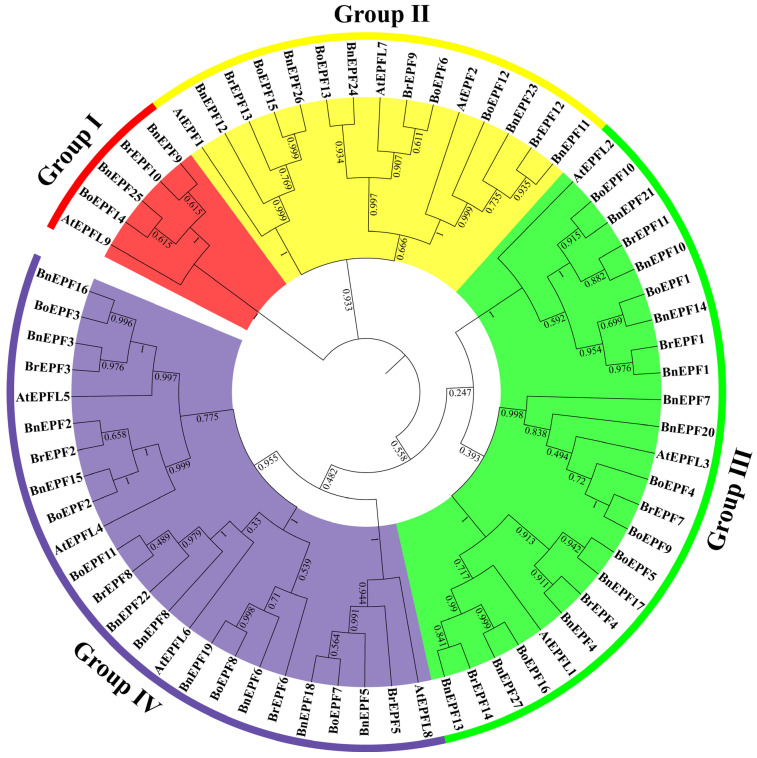
In this research, the phylogenetic tree of the EPF family is shown for *A. thaliana*, *B. napus*, *B. oleracea*, and *B. rapa*. The phylogenetic tree was constructed using the Maximum Likelihood approach in MEGA 11 software. The four main phylogenetic branches are marked with different backgrounds.

To examine the phylogenetic relationships among EPF proteins across diverse plant species, 11 *A. thaliana* EPF proteins, 27 *B. napus* EPF proteins, 14 *B. rapa* EPF proteins, and 16 *B. oleracea* EPF proteins were selected to generate a phylogenetic tree (Figure 2 and Supplementary File S4). The phylogenetic relationships among the EPF proteins were studied. The findings indicated that the EPF proteins clustered into four distinct clades (I, II, III, and IV). The distribution of the evolutionary clades varied significantly. The most abundant branches were Group III and Group IV, each with 24 members. Group III includes three AtEPFs, ten BnEPFs, five BrEPFs, and six BoEPFs, while Group IV includes four AtEPFs, ten BnEPFs, five BrEPFs, and five BoEPFs. Next was Group II, which has fifteen members, including three AtEPFs, five BnEPFs, three BrEPFs, and four BoEPFs. Group I had the fewest members, with one AtEPF, two BnEPFs, one BrEPF, and one BoEPF. It was worth noting that all branches contain EPF proteins from *B. napus*, *B. rapa, B. oleracea*, and *A. thaliana*. This distribution highlights their close genetic relationship.

### 3.4. Analysis of EPF Family Structure, Conserved Motifs, and Cis-Regulatory Elements in the Promoter

**Figure 3:**
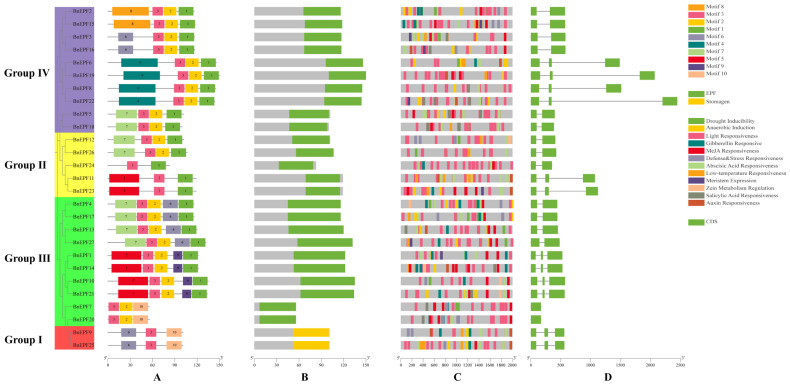
Analysis of the gene structure of the *EPF* family in *B. napus*. (**A**) Conserved motifs within the BnEPF family proteins. (**B**) Pfam domain structure of BnEPF family proteins. (**C**) Promoter cis-acting element of *BnEPFs*. (**D**) The mRNA structure of *BnEPFs*.

Studying motifs within conserved domains provides valuable insights into protein function, structure, and evolution. Conserved motif analysis reveals that Motif 3 was common in all BnEPF proteins; all BnEPF proteins in Group I contained Motif 6 and Motif 10; all BnEPFs in Group III contained Motif 2, with Motif 9 appearing in only some members of this group; and every BnEPF in Group IV contained Motif 1 and Motif 2, with Motif 4 and Motif 8 also present in only some members of this group (Figure 3A). The structural features of BnEPFs revealed that proteins in Groups I, II, and III all contain an EPF domain, while those in Group IV feature a Stomagen domain (Figure 3B). The examination of promoter cis-acting elements suggested that *BnEPFs* may have developed mechanisms to respond to stress and signals from plant hormones (Figure 3C). Notably, there were up to 342 light-responsive elements, significantly outnumbering other types of cis-acting elements. Additionally, *BnEPF5* was found to contain the highest number of cis-acting elements, totaling 42, suggesting that it is likely to have important roles in the growth and development of *B. napus*. The analysis of the mature mRNA structure of *BnEPFs* revealed that they contain between one and three coding sequence (CDS) regions (Figure 3D). However, no untranslated region (UTR) was detected in any of the *BnEPFs*.

### 3.5. Collinearity Analysis of EPF Gene Family

**Figure 4:**
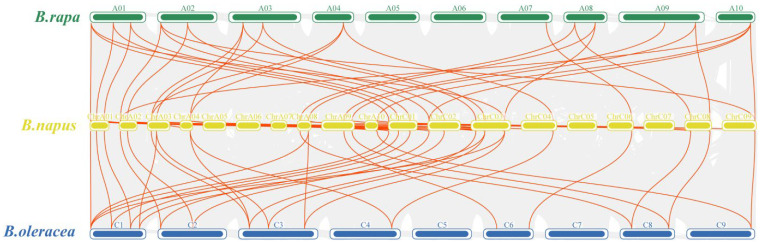
Synteny analysis of *EPF* genes in *B. rapa*, *B. napus*, and *B. oleracea*. The prefixes “*B. rapa*”, “*B. napus*”, and “*B. oleracea*” denote Brassica species, specifically *Brassica rapa*, *B. napus*, and *B. oleracea*, respectively. The gray lines in the background represent duplication events within the genomes of *B. rapa*, *B. napus*, and *B. oleracea*, whereas the red lines indicate syntenic pairs of *EPF* genes. The chromosomes of *B. rapa*, *B. napus*, and *B. oleracea* are represented by green, yellow, and blue bars, respectively. Each chromosome is labeled with its respective number at the top.

To uncover homologous gene functions and phylogenetic relationships across species, a collinearity analysis among *EPFs* in *B. rapa, B. napus*, and *B. oleracea* was conducted. The findings indicated that the *EPFs* of *B. napus* exhibited 32 and 31 pairs of homologous genes with the *EPFs* of *B. rapa* and *B. oleracea*, respectively (Figure 4 and Supplementary File S5). *BnEPF23* did not exhibit collinearity with the *EPF* genes of either species.

**Figure 5:**
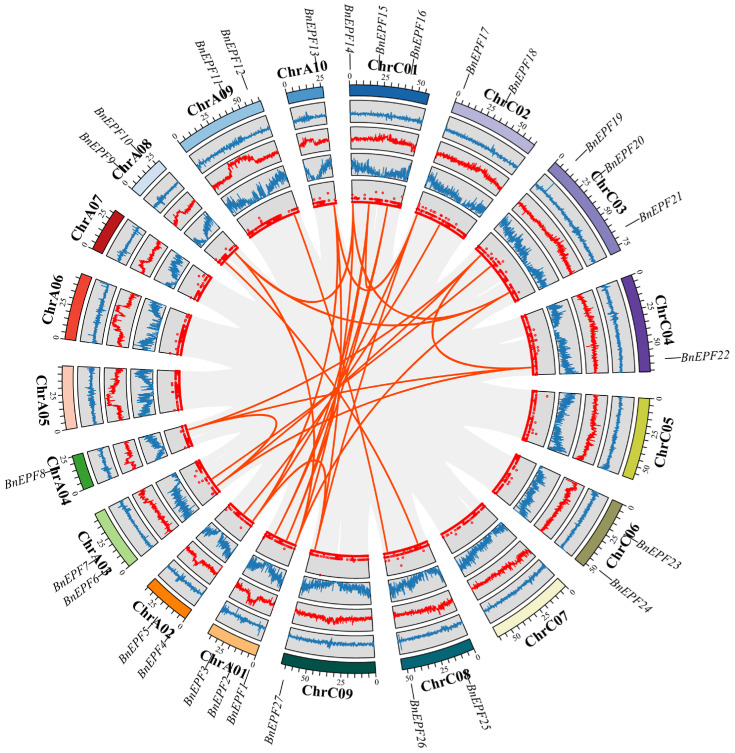
Synteny analysis among *BnEPFs*. Gray lines denote all synteny blocks between each chromosome, while thick red lines indicate duplicated pairs of *EPFs*. The circles in this Figure represent, from innermost to outermost, the ratio of unknown bases (N ratio), gene density, GC ratio, GC skew, and chromosome length of the *B. napus* genome. Chromosome names are labeled at the bottom of each chromosome, and the positions of *BnEPFs* are indicated accordingly.

To enhance our understanding of the evolutionary relationships among *BnEPFs* in *B. napus*, we analyzed gene duplication events. The findings reveal that there were 27 segmental duplication events identified in the genome of *B. napus*, involving 13 *BnEPFs*. These duplicated segments were located on chromosomes ChrA01, ChrA02, ChrA03, ChrA04, ChrA08, ChrA09, ChrA10, ChrC01, ChrC02, ChrC03, ChrC04, ChrC08, and ChrC09 (Figure 5 and Supplementary File S6). Among them, there were no collinear relationships between *BnEPF11, BnEPF23,* and *BnEPF24* with other *BnEPFs*.

### 3.6. Prediction of miRNA Targeting Relationships with BnEPFs

**Figure 6:**
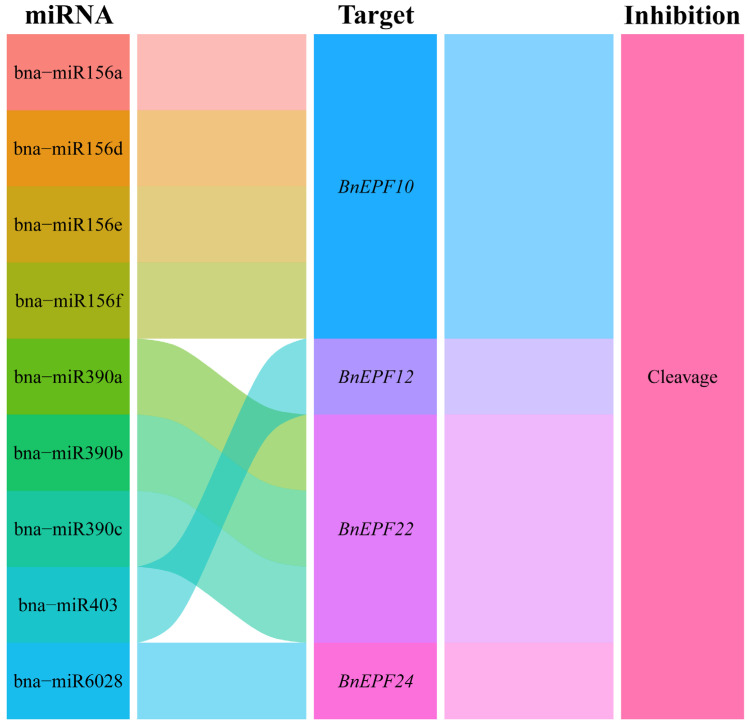
Sankey diagram depicting the relationships between miRNA targeting and *BnEPFs* transcripts. The diagram consists of three columns representing miRNA, *BnEPFs*, and the inhibition effect.

As illustrated in Figure 6, a total of nine miRNAs originating from *B. napus* were identified to target four *BnEPF* genes through cleavage. No miRNAs were found to exert translation inhibition effects on the *BnEPFs*. These results suggested that several miRNAs played a role in the post-transcriptional regulation of *BnEPFs* by specifically targeting them for cleavage.

### 3.7. Examination of Transcriptome Expression Profiles of BnEPFs across Various Exogenous Hormone Treatments and Abiotic Stress Conditions

**Figure 7:**
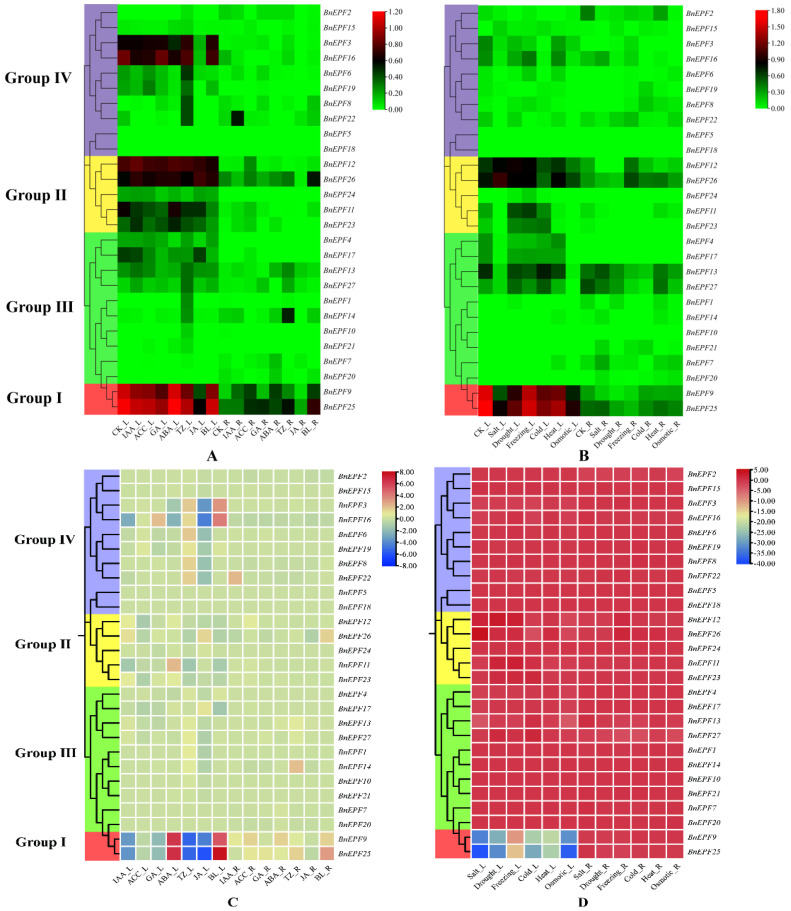
Study of *BnEPFs*’ expression profiles across diverse treatments. (**A**) Hormone treatments (CK, IAA, ACC, GAs, ABA, TZ, JA, and BL); (**B**) Non-biological stress treatments (CK, salt, drought, freezing, cold, heat, and osmotic); (**C**) Hormone treatment (difference between treatment and CK); (**D**) Non-biological stress treatments (difference between treatment and CK). L: leaves; R: Roots.

To investigate the patterns of *BnEPFs* under various hormone treatments (Control (CK), IAA (10 μmol·L^−1^ growth hormone), ACC (10 μmol·L^−1^ ethylene), GAs (10 μmol·L^−1^ gibberellin), ABA (10 μmol·L^−1^ abscisic acid), TZ (10 μmol·L^−1^ trans-zeatin), JA (10 μmol·L^−1^ jasmonic acid), and BL (10 μmol·L^−1^ brassinolactone), measured after 6 h) and abiotic stresses (Control (CK), salt (200 mmol·L^−1^ NaCl), drought (exposure to airflow for 1 h), freezing (recovery to 25 °C after 3 h at −4 °C), cold (4 °C), heat (recovery to 25 °C after 3 h at 38 °C), and osmotic stress (300 mmol·L^−1^ mannitol) measured after 24 h), we downloaded the TPM data for *BnEPFs* of *B. napus* ZS11 from the BnIR website under these various conditions. The data, after calculating log_10_ (TPM + 1), were used to generate the gene expression heatmap (Figure 7A,B). The results showed that *BnEPFs* in Group I generally exhibited high expression in leaves under various plant hormone treatments and abiotic stresses, with partial responses to plant hormone signals in roots, but lower expression under different abiotic stresses. In Group II, *BnEPF12* and *BnEPF26* consistently demonstrated elevated expression levels in leaves across all treatments and were partially expressed in roots; *BnEPF11* and *BnEPF23* exhibited partial responsiveness to environmental changes in leaves across different treatments, whereas their expression in roots was either absent or minimal; and *BnEPF24* exhibited low expression under all conditions. In Group III, *BnEPF13* and *BnEPF27* were partially expressed in both leaves and roots under various treatments, *BnEPF4* and *BnEPF17* were partially expressed in leaves, while other *BnEPFs* either did not express or expressed modestly. In Group IV, *BnEPF3* and *BnEPF16* were expressed abundantly in leaves under different plant hormone treatments, *BnEPF5* and *BnEPF18* were not expressed under any conditions, and other *BnEPFs* showed partial expression in both leaves and roots under different experimental conditions.

We plotted a heatmap of the differences between *BnEPFs* under various treatments and the control (Figure 7C,D). The results showed that most *BnEPFs* did not exhibit significant upregulation or downregulation under hormone treatments. However, members of Group I in the L part displayed a trend of upregulation or downregulation in response to all hormones except ACC and GA. *BnEPF3* and *BnEPF16* in the L part might be upregulated or downregulated in response to hormone treatments. Under abiotic stress treatments, most *BnEPFs* showed no significant changes or slight downregulation, while members of Group I in the L part displayed a notable downregulation trend.

### 3.8. Analysis of BnEPF Expression under Salt Stress

**Figure 8:**
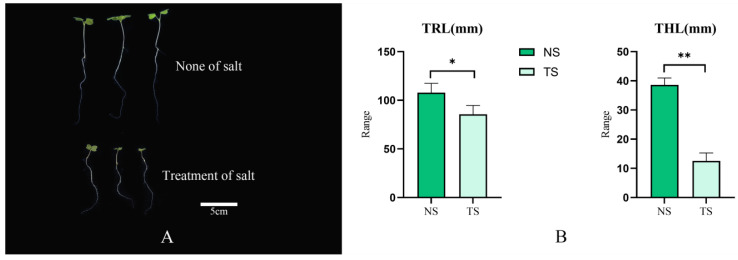
Phenotypic identification and analysis of *B. napus.* (**A**) Impact of salt absence and salt treatment on the seedling growth of *B. napus*. (**B**) Under no salt (NS) and treatment of salt (TS) conditions, the root morphology indices and hypocotyl morphology indices of *B. napus* changed. TRL: Total root length; THL: Total hypocotyl length; NS: No salt, TS: Treatment of salt. *: significant differences between treatments at *p* ≤ 0.05. **: significant differences between treatments at *p* < 0.01.

The *EPF* family is pivotal in regulating stomatal development in plants, which, in turn, is closely linked to plant responses to salt stress. Therefore, studying the expression and physiological role of the *EPF* family in rapeseed may help gain insights into the mechanisms of the adaptation of rapeseed to salt stress and provide references for the breeding of salt-tolerant rapeseed. Therefore, the influence of *BnEPF* genes on the salt stress response in rapeseed (Jia 917, Provided by the National Rapeseed Engineering Center of Huazhong Agricultural University) were studied under two salt treatment conditions as follows: non-salt (NS: NaCl: 0%) and salt treatment (TS: NaCl: 0.8%). The results indicate that TS significantly inhibits the growth of rapeseed leaves. We noted that rapeseed responds to the adverse conditions induced by TS by altering the physical appearance of its leaves and roots (Figure 8A). Analysis under no salt (NS) and salt treatment (TS) conditions indicated that TS significantly inhibited both the aboveground and underground growth of rapeseed (*B. napus* L.) (Figure 8B). Compared to NS, TS significantly inhibited the changes in the total root length (TRL) and total hypocotyl length (THL) of rapeseed. This indicates that under TS conditions, the growth of rapeseed is hindered. This hindrance is attributed to reduced stomatal function and water management, which help the plant adapt to salt stress conditions, but may also lead to smaller plant growth. Some *BnEPFs* were detected via qPCR, suggesting their potential involvement in regulating abiotic stress tolerance in rapeseed. The findings indicate that salt treatment significantly inhibits the growth of rapeseed leaves.

**Figure 9:**
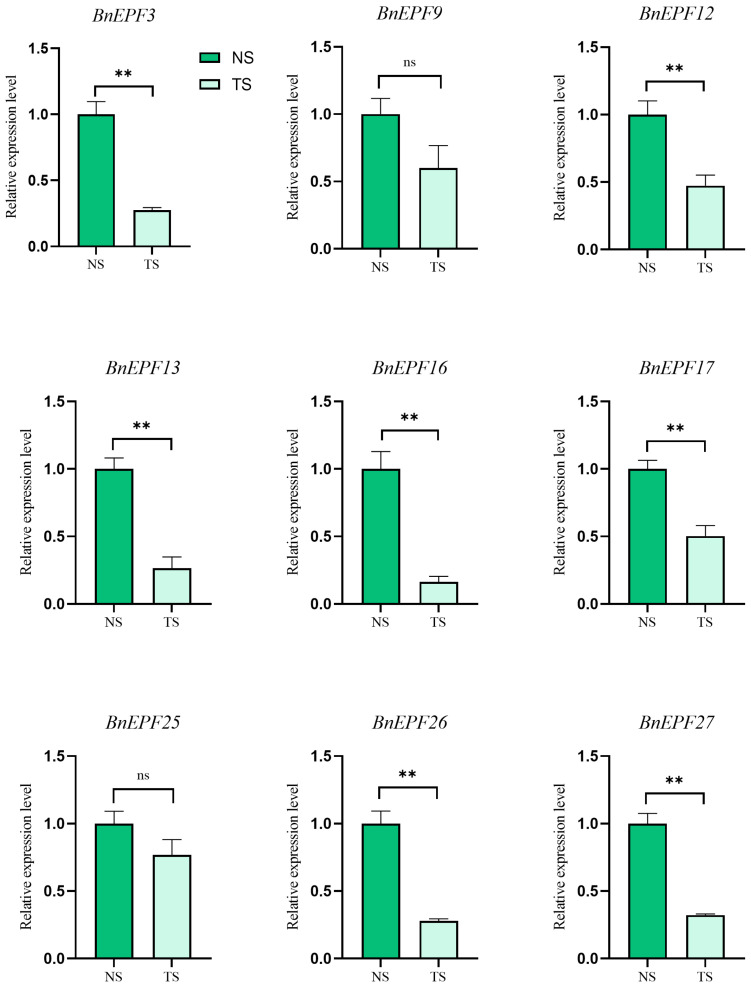
Non-biological stress-induced expression patterns of *BnEPFs*. The *y*-axis indicates relative expression levels, calculated using the 2^−∆∆Ct^ method. The *x*-axis represents various treatments of rapeseed. The expression profiles of *BnEPFs* were obtained under no salt (NS: NaCl: 0%) and treatment of salt (TS: NaCl: 0.8%) levels. NS: No salt, TS: Treatment of salt. ns: no significant difference. **: significant differences between treatments at *p* < 0.01.

The results indicate that, WHEN compared to the condition without salt stress (NS), the treatment of salt (TS) CONDITION resulted in a significant downregulation of *BnEPF3*, *BnEPF12*, *BnEPF13*, *BnEPF16*, *BnEPF17*, *BnEPF26*, and *BnEPF27*. Additionally, there was also an observed downward trend in the expression of *BnEPF9* and *BnEPF25*; however, the observed difference did not reach statistical significance (Figure 9). Overall, the response of *BnEPFs* to salt stress exhibited a general downregulation trend, suggesting that they may affect stomatal function and signal transduction processes to enhance rapeseed resistance to TS conditions.

## 4. Discussion

*EPFs*, small secreted peptides present across various plant species, are essential for regulating plant growth, development, and responses to stress. Recent developments in sequencing technologies have facilitated the discovery of a growing number of *EPF* genes across diverse plant species, including poplar, *Arabidopsis*, tomato, and rice. [17,40,41]. These genes help reduce water loss, maintain water balance within the plant, and enhance the plant’s ability to adapt to salt stress through signal transduction and gene expression regulation. Rapeseed, a globally significant oil crop, holds significant value. Despite the publication of the reference genome of *B. napus* [42], there is still a need for comprehensive research on the *EPF* gene family in rapeseed. 

Previous studies have identified 25 potential *EPF* genes in the *B. napus* cultivar Darmor-bzh genome using BLAST methods and have conducted the detailed functional analyses of *EPF2*. However, the subsequent analyses of other *EPF* subfamilies using bioinformatics and molecular biology approaches have not been performed [43]. This research represents the first instance of identifying and analyzing the *BnEPF* gene family in rapeseed at the whole-genome level. The findings indicated that the *BnEPF* gene family members in rapeseed are more num erous than those in *A. thaliana* (11 members), *B.rapa* (14 members), and *B. oleracea* (16 members). All members of the BnEPF family contained complete conserved domains. Motif 3 was present in all BnEPFs; all BnEPF proteins in Group I contained Motif 6 and Motif 10; all *BnEPFs* in Group III contained Motif 2, with Motif 9 appearing in only some members of this group; and each BnEPF protein in Group IVfeatures Motif 1, Motif 2, Motif 4 was present only in some members of this group. The presence of different conserved motifs may indicate functional differences among members of different groups. *EPF/EPFL* peptides, as a class of peptide hormones, generally acted as extracellular ligands at the top of various developmental pathways, requiring the activation of downstream cascades through binding to surface receptor kinases on the cell membrane to exert their effects [44]. This study found that members of the *BnEPF* family are primarily located in the extracellular matrix, indicating that they may exert their effects through signal transduction and gene expression regulation.

In the process of species evolution, homologous genes often retain similar functions [45,46]. Based on the evolutionary analysis of protein sequences from *A. thaliana, B. napus, B. rapa*, and *B. oleracea*, *BnEPFs* were unevenly distributed across four clades. The distribution of BnEPF proteins across each clade revealed that Group II had the smallest share, comprising only 33.3% of the total, while the proportions in Group III and Group IV were tied for the highest at 41.7% (Figure 2). This suggests that the Group II clade in *B. napus* exhibits greater conservation in gene evolution relative to *A. thaliana*, *B. rapa*, and *B. oleracea*, although gene family duplication events may have occurred in the *B. napus* genome within the other three clades. The analysis of the gene structure and conserved domains of *BnEPFs* indicated their evolutionary conservation across species, including evident conservation in *Sorghum bicolor* [47].

Plants have evolved various mechanisms to defend themselves against biotic and abiotic challenges, primarily manifested in the expression of genes responsive to these stimuli. Transcription factors govern the activity of these genes, emphasizing the critical role of analyzing promoter cis-acting elements in comprehending the function of specific genes [48]. In this study, it was found that the upstream regions of most *BnEPF* genes contain elements that are responsive to light, totaling 342, which is significantly higher than any other cis-acting elements. Light is a fundamental element in controlling plant growth, development, and many important biological processes [49]. These light-responsive elements form a foundation for understanding the role of this gene family in light-regulated growth and development, implying that this gene family may participate in light regulation and potentially influence the growth and development of rapeseed.

Studies indicate that *EPF* genes are associated with various abiotic stresses, but their expression patterns vary across different species. The increased expression of the *TaEPF1B* gene in wheat can reduce stomatal density and improve water use [50]. In rice, the heightened expression of the homologous *EPFL1* promoted internode growth, while the specific splicing of the RAE2 gene in rice spikelets facilitated the extension of the mature RAE2 peptide mediated by *SLP1* [18]. In this study, qRT-PCR analysis revealed that under salt stress conditions, all nine *BnEPF* genes in rapeseed showed a downregulation trend. *BnEPFs* may regulate the closure of rapeseed stomata in this way to cope with the adverse effects of salt stress on its growth. This indicates that the *EPF* gene family plays a crucial role in plant adaptation to and resilience in salt-challenged environments by regulating gene activity, stomatal function, and signal transduction pathways.

## 5. Conclusions

Twenty-seven rapeseed *EPF* genes were discovered, spanning fourteen chromosomes. The rapeseed *EPF* genes were divided into four groups, with those in the same group displaying similar gene and protein structures. The activity of *BnEPF* genes increased under salt stress, suggesting their involvement in the plant’s response to saline conditions.

## Figures and Tables

**Table 1:** Primer sequence information.

Gene Name	Forward	Reverse
*BnEPF3*	GCTCCCACCGTCGTTTCA	TGACCCACCTGCCTCCTT
*BnEPF9*	TGATTGGGTCGACGGCAC	TGCCTTCAACGGGGACTTG
*BnEPF12*	GCATTTTCCGACGCACCC	CACAAGACGGCATGGGGA
*BnEPF13*	CAAGTCCCCACTCTGCCG	GGGAGGAAGGAGGCCGTA
*BnEPF16*	TGCTCCCACCGTCGTTTC	GAGGCGGTTGCGGAGTAG
*BnEPF17*	CCGAGCTGCCACAACAGA	GGGAGGAAGGAGGCCGTA
*BnEPF25*	TGATTGGGTCGACGGCAC	TGCCTTCAACGGGGACTTG
*BnEPF26*	GTCTAGGCTGCCGGACTG	GCGATGCACACACGAAGC
*BnEPF27*	CTACACCACCGAGCTGCC	GGGAGGAAGGAGGCCGTA
*Actin*	TGGGTTTGCTGGTGACGAT	TGCCTAGGACGACCAACAATACT

## Data Availability

All the data generated or analyzed during this study are included in this published article and its Supplementary Information Files.

## Supplementary Materials

The following supporting information can be downloaded at https://www.mdpi.com/article/10.3390/genes15070912/s1, Supplementary File S1: Detail information of *B. napus EPF* gene family. Supplementary File S2: *EPFs* amino acid sequences in *B. napus*, *B. rapa*, *B. oleracea*, and *A. thaliana*. Supplementary File S3: Predicted secondary structure of BnEPF proteins. Supplementary File S4: Number of EPF proteins contained in each group in the phylogenetic tree. Supplementary File S5: Homologous gene pairs among *EPF* genes of *B. napus*, *B. rapa*, and *B. oleracea*. Supplementary File S6: Gene duplication of *BnEPFs* in *B. napus*.
