# Supplementary material for: Comprehensive Identification and Expression Profiling of Epidermal Pattern Factor (EPF) Gene Family in Oilseed Rape (Brassica napus L.) under Salt Stress
Source: Genes (Basel). 2024 Jul 12;15(7):912. doi: 10.3390/genes15070912 (PMC11275378; doi:10.3390/genes15070912)
Supplement: Supplementary file 1 [file genes-15-00912-s001.zip › Supplementary File S1.pdf]

Supplementary File S1. Detail information of *B. napus EPF* gene family.

Gene Name	Gene ID	Chromosome	Protein length (aa)	Molecular Weight (KDa)	Isoelectric Point	GRAVY	Subcellular Localization
BnEPF1	*BnaA01G0012000ZS*	A01	122	13.58	8.45	-0.47	Extracellular
BnEPF2	*BnaA01G0203200ZS*	A01	116	12.74	10.15	-0.22	Extracellular
BnEPF3	*BnaA01G0298800ZS*	A01	117	12.63	9.65	-0.09	Extracellular
BnEPF4	*BnaA02G0035800ZS*	A02	116	12.79	9.24	-0.09	Extracellular
BnEPF5	*BnaA02G0246000ZS*	A02	102	11.81	6.88	-0.45	Extracellular
BnEPF6	*BnaA03G0146500ZS*	A03	146	16.06	9.51	-0.29	Extracellular
BnEPF7	*BnaA03G0335800ZS*	A03	56	6.25	7.59	-0.72	Nuclear
BnEPF8	*BnaA04G0196100ZS*	A04	145	15.98	9.79	-0.23	Extracellular
BnEPF9	*BnaA08G0057000ZS*	A08	101	11.77	9.27	-0.32	Extracellular
BnEPF10	*BnaA08G0188500ZS*	A08	135	15.19	8.93	-0.34	Extracellular
BnEPF11	*BnaA09G0378400ZS*	A09	119	13.17	9.10	-0.07	Extracellular
BnEPF12	*BnaA09G0602900ZS*	A09	102	11.42	9.05	0.01	Extracellular
BnEPF13	*BnaA10G0243600ZS*	A10	120	13.29	8.62	-0.22	Extracellular
BnEPF14	*BnaC01G0014200ZS*	C01	122	13.62	8.84	-0.39	Extracellular
BnEPF15	*BnaC01G0255100ZS*	C01	118	12.97	10.25	-0.22	Extracellular
BnEPF16	*BnaC01G0368200ZS*	C01	117	12.69	9.50	-0.04	Extracellular
BnEPF17	*BnaC02G0040000ZS*	C02	116	12.80	9.42	-0.03	Extracellular
BnEPF18	*BnaC02G0329600ZS*	C02	100	11.53	8.71	-0.42	Extracellular
BnEPF19	*BnaC03G0170600ZS*	C03	150	16.62	9.53	-0.35	Extracellular
BnEPF20	*BnaC03G0403000ZS*	C03	56	6.25	7.59	-0.72	Nuclear
BnEPF21	*BnaC03G0685300ZS*	C03	134	15.05	8.78	-0.30	Extracellular
BnEPF22	*BnaC04G0499900ZS*	C04	144	15.76	9.67	-0.28	Extracellular
BnEPF23	*BnaC06G0101300ZS*	C06	119	13.22	8.99	-0.10	Extracellular
BnEPF24	*BnaC06G0384600ZS*	C06	83	8.85	8.47	0.50	Extracellular
BnEPF25	*BnaC08G0080100ZS*	C08	101	11.70	9.12	-0.29	Extracellular
BnEPF26	*BnaC08G0457000ZS*	C08	107	12.00	9.20	-0.01	Extracellular
BnEPF27	*BnaC09G0554600ZS*	C09	132	14.73	9.06	-0.20	Extracellular
