# Supplementary material for: Comprehensive Identification and Expression Profiling of Epidermal Pattern Factor (EPF) Gene Family in Oilseed Rape (Brassica napus L.) under Salt Stress
Source: Genes (Basel). 2024 Jul 12;15(7):912. doi: 10.3390/genes15070912 (PMC11275378; doi:10.3390/genes15070912)
Supplement: Supplementary file 1 [file genes-15-00912-s001.zip › Supplementary File S3.pdf]

Supplementary File S3. Predicted secondary structure of *BnEPF* proteins.

Protein Name	Alpha helix (Hh)	Extended strand (Ee)	Random coil (Cc)	Protein Name	Alpha helix (Hh)	Extended strand (Ee)	Random coil (Cc)
BnEPF1	15.57%	15.57%	68.85%	*BnEPF15*	27.97%	7.63%	64.41%
BnEPF2	25.86%	9.48%	64.66%	*BnEPF16*	21.37%	10.26%	68.38%
BnEPF3	21.37%	10.26%	68.38%	*BnEPF17*	13.79%	12.07%	74.14%
BnEPF4	15.52%	13.79%	70.69%	*BnEPF18*	23.00%	12.00%	65.00%
BnEPF5	25.49%	11.76%	62.75%	*BnEPF19*	16.67%	7.33%	76.00%
BnEPF6	19.86%	8.90%	71.23%	*BnEPF20*	1.79%	19.64%	78.57%
BnEPF7	1.79%	19.64%	78.57%	*BnEPF21*	20.90%	11.94%	67.16%
BnEPF8	14.48%	6.90%	78.62%	*BnEPF22*	14.58%	7.64%	77.78%
BnEPF9	25.74%	16.83%	57.43%	*BnEPF23*	23.53%	15.13%	61.34%
BnEPF10	22.22%	9.63%	68.15%	*BnEPF24*	21.69%	20.48%	57.83%
BnEPF11	21.01%	14.29%	64.71%	*BnEPF25*	22.77%	17.82%	59.41%
BnEPF12	7.84%	12.75%	79.41%	*BnEPF26*	13.08%	10.28%	76.64%
BnEPF13	13.33%	10.00%	76.67%	*BnEPF27*	17.42%	6.06%	76.52%
BnEPF14	20.49%	11.48%	68.03%				
