# Supplementary material for: Comprehensive Identification and Expression Profiling of Epidermal Pattern Factor (EPF) Gene Family in Oilseed Rape (Brassica napus L.) under Salt Stress
Source: Genes (Basel). 2024 Jul 12;15(7):912. doi: 10.3390/genes15070912 (PMC11275378; doi:10.3390/genes15070912)
Supplement: Supplementary file 1 [file genes-15-00912-s001.zip › Supplementary File S4.pdf]

Supplementary File S4. Number of *EPF* proteins contained in each group in the phylogenetic tree.

Group	Group I	Group II	Group III	Group IV
AtEPF Number	1	3	3	4
BnEPF Number	2	5	10	10
BrEPF Number	1	3	5	5
BoEPF Number	1	4	6	5
